# In Vitro Prebiotic Effects of Malto-Oligosaccharides Containing Water-Soluble Dietary Fiber

**DOI:** 10.3390/molecules25215201

**Published:** 2020-11-09

**Authors:** Eun Yeong Jang, Ki-Bae Hong, Yeok Boo Chang, Jungcheul Shin, Eun Young Jung, Kyungae Jo, Hyung Joo Suh

**Affiliations:** 1 Department of Integrated Biomedical and Life Science, Graduate School, Korea University, Seoul 02841, Korea; eusilverl@ckdhc.com (E.Y.J.); oobkoey@gmail.com (Y.B.C.); 2 Department of Food Science and Biotechnology, Dongguk University, Goyang 10326, Korea; kibae.hong@gmail.com; 3 Department of R&D, Neo Cremar Co., Ltd., Seoul 05702, Korea; sjc7254@cremar.co.kr; 4 Department of Home Economic Education, Jeonju University, Jeonju 55069, Korea; jjj@jj.ac.kr

**Keywords:** malto-oligosaccharide, SCFA, in vitro fermentation, prebiotics

## Abstract

This study measured the proliferative activity of malto-oligosaccharide (MOS) as a prebiotic against Bifidobacteria, resistance to digestion in vitro, and changes during in vitro fermentation by human fecal microorganisms. It consisted of 21.74%, 18.84%, and 11.76% of maltotriose, maltotetraose, and maltopentaose produced by amylase (HATT), respectively. When 1% of MOS was added to a modified PYF medium as the carbon source, proliferation of *Bifidobacterium breve* was increased significantly. During the in vitro digestion test, MOS was partially degraded by intestinal enzymes. Fermentation characteristics by human fecal microorganisms were evaluated by adding 1% galacto-oligosaccharide (GOS), as well as 1% and 2% MOS as carbon sources to the basal medium, respectively. In comparison with the addition of 1% of MOS and GOS, the total short chain fatty acid (SCFA) content increased over time when 2% of MOS was added. The species diversity and richness of intestinal microbiota increased significantly with 2% MOS compared to those with 1% GOS. In addition, the 2% addition of MOS reduced intestinal pathobiont microorganisms and increased commensal microorganisms including *Bifidobacterium* genus. Collectively, MOS produced by amylase increased the SCFA production and enhanced the growth of beneficial bacteria during in vitro fermentation by human fecal microbiota.

## 1. Introduction

With economic development and a sufficient supply of nutrients, human interest has shifted from survival to maintaining a healthy life. Therefore, improving human health by regulating the microbiome is presently emerging as an important approach and strategy. Prebiotics are substrates that are selectively utilized by host microorganisms containing non-carbohydrate substances to promote the beneficial function in the gut. Prebiotics are selectively utilized for changes in the beneficial taxa and microbial ecosystems that maintain, improve, and restore host health [1,2,3]. Nondigestible oligosaccharides such as galacto-oligosaccharide (GOS) and fructo-oligosaccharide (FOS) are known to improve host health by selectively stimulating the growth and activity of intestinal microorganisms [4,5].

Among the oligosaccharides, malto-oligosaccharide (MOS) is a saccharide in which a glucose molecule forms one or more branched bonds (α-1,4) for degree of polymerization (DP) 2–9 [6]. MOS is a functional oligosaccharide that can be used in the food, beverage, cosmetic, and pharmaceutical industries due to its mild sweetness, relatively low osmotic pressure, high moisturizing power, and moderate viscosity [7,8]. When added to starchy food, MOS inhibits aging by interfering with the binding of amylopectin. Furthermore, MOS regulates blood sugar and cholesterol levels and relieves constipation [9,10].

MOS prepared using only α-amylase without pullulanase treatment contains undegraded amylopectin; therefore, it is presumed to have a prebiotic activity as it is resistant to α-amylase degradation and could selectively stimulate the growth and function of intestinal microbes [11]. Moreover, resistant starch that is not degraded to α-amylase has prebiotic properties [12]. Resistant starch, which is difficult to decompose by digestion, stimulates intestinal microbial activity in order to improve the intestinal environment and maintain intestinal integrity. Indigestible oligosaccharides with a short chain length (2–10) are also known to have a prebiotic effect due to selective metabolism [13,14]. Many studies on the relationship between prebiotics and the intestinal microbiota have been done mainly in animal models and in vitro models with pure cultures. However, these experimental methods are limited because of the differences in population groups in the microbiota. In addition, fecal strains behave differently in a mixed culture than in pure culture [15,16]. Mixed fermentation systems are more effective and realistic in evaluating polysaccharides in the human gut microbiota. Hence, in vitro fermentation by human feces has been widely used to study the function and metabolism of food components such as polysaccharides [17].

In order to evaluate the potential activity of MOS produced by α-amylase as a prebiotic, it is necessary to evaluate the effect of intestinal microbiota through in vitro fermentation by the mixed fecal microorganisms of human origin. Therefore, in the present study, proliferation of *Bifidobacterium* and digestibility of MOS were measured in vitro to evaluate the effects of MOS as a prebiotic. In addition, in vitro fermentation using human fecal bacteria was performed to evaluate fermentation characteristics including changes in the content of SCFA and changes in intestinal microorganisms.

## 2. Results

### 2.1. MOS Content after Differences in the Enzyme and Treatment Process

For MOS production, the productivity was measured according to the liquefaction and saccharification processes. Liquefaction and saccharification enzymes for MOS production were selected by considering the production cost reduction and process simplification. Moreover, medium-temperature α-amylase (MTAA) and high-temperature α-amylase (HTAA) were selected as process enzymes based on their reaction temperatures. For liquefaction and saccharification, commercial amylases such as MTAA and Fungamyl 800L were used, respectively, whereas α-amylase (HTAA) was used to react without distinguishing between liquefaction and saccharification (Table 1). When liquefaction and saccharification were conducted with different enzymes, the MOS content after 20 h of saccharification was 35.56%. In contrast, when treated with HTAA alone, the MOS content at the same time period was 52.34%. Thus, we confirmed that it is more suitable to perform the liquefaction and saccharification processes using only the high-temperature enzyme HTAA than adding the liquefaction and saccharification enzymes to produce MOS. MOS produced by HTAA, a thermophilic α-amylase, comprises 48.8% dietary fiber, and 99% of the dietary fiber was composed of soluble fiber (data not shown).

### 2.2. Proliferation Effect of MOS on Bifidobacterium sp.

**Figure 1:**
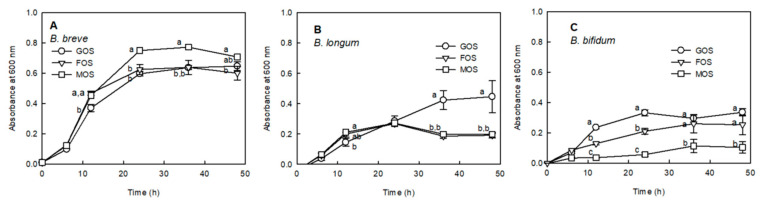
Proliferation effect of fructo-oligosaccharide (FOS), galacto-oligosaccharide (GOS), and malto-oligosaccharide (MOS) on *Bifidobacterium breve* ATCC 15,700 (**A**), *Bifidobacterium longum* ATCC 15,707 (**B**), and *Bifidobacterium bifidum* ATCC 3357 (**C**). The strains were cultured by adding 1% of GOS, FOS, or MOS to the modified peptone yeast extract fructose (PYF) medium without a carbon source. Data are expressed as the mean ± standard deviation, and different letters indicate significant differences at *p* < 0.05 between groups at the same fermentation time.

To measure the proliferation of Bifidobacteria by MOS, the proliferation activities of FOS and GOS, known as substrates for the selective growth of *Bifidobacterium* sp., were compared to that of MOS (Figure 1).

MOS revealed a superior effect on the proliferation of *Bifidobacterium breve* compared to that of GOS and FOS from 24 h after anaerobic fermentation (Figure 1A: *p* < 0.05). Conversely, MOS caused a considerably lower proliferation of *Bifidobacterium bifidum* than FOS and GOS (Figure 1C). Moreover, MOS caused a reasonably reduced proliferation of *Bifidobacterium longum* compared to GOS, whereas the proliferative activity was similar to that of FOS (Figure 1B). *B. breve* exhibited a higher cell growth rate when utilizing MOS in comparison with the existing *Bifidobacterium* growth factors GOS and FOS after 24 h of culture, whereas MOS showed a proliferative activity similar to that of FOS in *B. longum*.

### 2.3. Effects of the Digestion Process on the Sugar Composition of MOS

**Figure 2:**
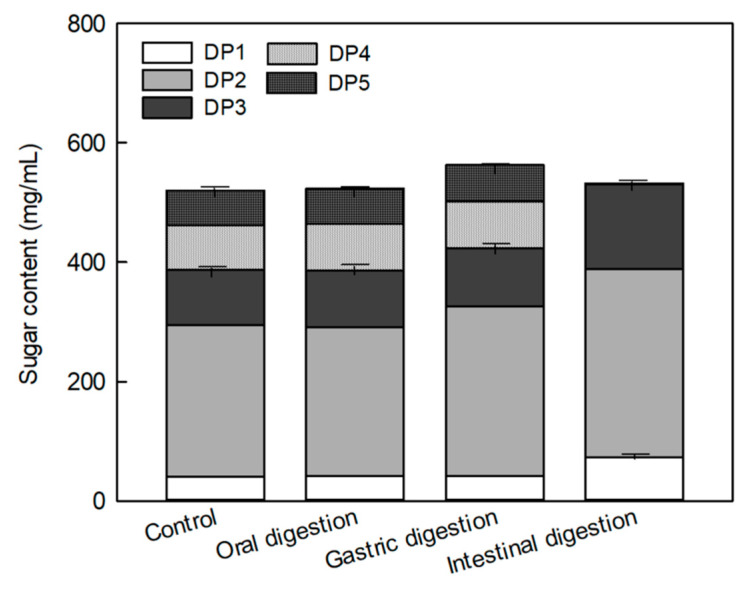
Changes in the sugar composition of malto-oligosaccharide (MOS) by in vitro digestion. In vitro digestion was conducted using salivary amylase, pepsin, and pancreatic enzymes. Data are expressed as the mean ± standard deviation. DP1: Glucose, DP2: Maltose, DP3: Maltotriose, DP4: Maltoteterose, DP5: Maltopentaose, Control: MOS without enzyme treatments.

The digestibility of MOS was determined using an in vitro digestion model. The change in the MOS constituent sugar was measured after treating each group with enzymes that digest carbohydrates in the mouth, stomach, and small intestine (Figure 2). No significant difference was observed in the total MOS (maltotriose (DP3) + maltotetraose (DP4) + maltopentaose (DP5)) content between before (control: MOS without the enzyme treatment) and after in vitro salivary and gastric digestions (Figure 2). Moreover, no significant increase was observed in the glucose and maltose contents, which were the decomposed products of MOS, after salivary and gastric enzyme treatments. However, the contents of glucose (DP1) and DP3 were significantly increased after the pancreatic enzyme treatment, whereas the contents of DP4 and DP5 significantly decreased from 78.78 and 60.67 mg/mL before the pancreatic enzyme treatment to 1.16 and 0.42 mg/mL after the treatment, respectively. The decrease in the DP4 and DP5 contents was found to increase the contents of DP1, maltose (DP2), and DP3. As DP4 and DP5 were degraded by small intestinal enzymes, the amount of total MOS was also decreased.

### 2.4. Effects of MOS on the Sugar Content and SCFA Production during Anaerobic Fermentation

The addition of GOS and MOS to the basal medium induced changes in the pH and reduced sugar, respectively, during anaerobic fermentation. The pH and reducing contents tended to decrease as the fermentation time increased (Figure S1).

**Figure 3:**
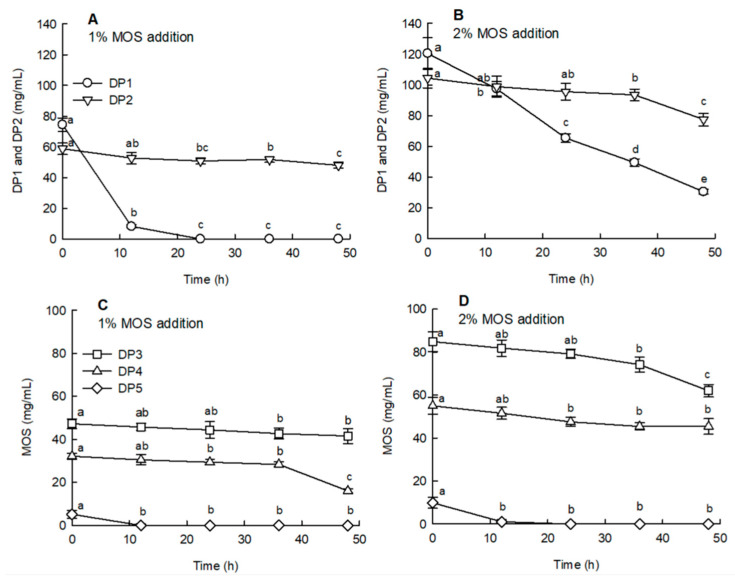
Changes in the sugar content of malto-oligosaccharide (MOS) by in vitro fermentation. In vitro fermentation was carried out at 37 °C by inoculating feces under anaerobic conditions. Anaerobic fermentation was performed by adding 1% MOS (**A**,**C**) and 2% MOS (**B**,**D**) to the basal medium. MOS includes maltotriose (DP3), maltotetrose (DP4), and maltopentaose (DP5). Data are expressed as the mean ± standard deviation, and different letters indicate significant differences in the content of the components before and during fermentation at *p* < 0.05.

Figure 3 illustrates the changes in the sugar content of MOS during anaerobic fermentation. When 1% and 2% MOS were added, the glucose (DP1) content tended to decrease rapidly with the increasing fermentation time (Figure 3A,B). DP5 was also decomposed and decreased when fermentation commenced (Figure 3C,D: *p* < 0.05). The DP2, DP3, and DP4 contents tended to decrease relatively slowly compared to those of DP1. However, in the case of DP4, the decrease was higher after 36 h of fermentation than before this time period (Figure 3C: *p* < 0.05). This indicated that DP2–DP4 gradually decreased as sugars with a high polymerization degree were converted to a low polymerization degree by the fermentation strains.

**Figure 4:**
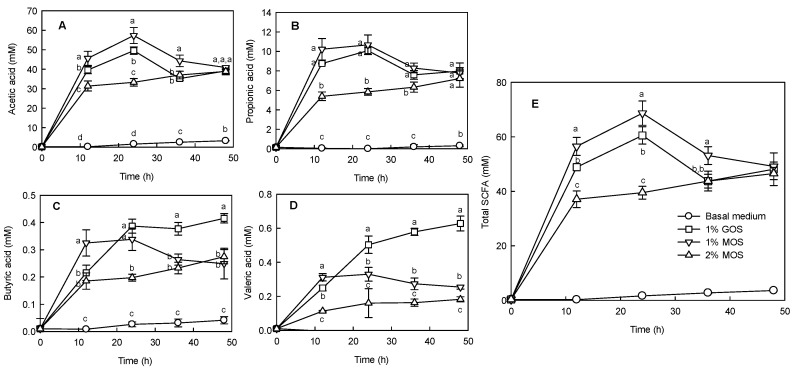
Changes in acetic acid (**A**), propionic acid (**B**), butyric acid (**C**), valeric acid (**D**), and total short chain fatty acid (SCFA, (**E**)) content during in vitro fermentation after the glucose and malto-oligosaccharide (MOS) addition. In vitro fermentation was carried out at 37 °C by inoculating feces under anaerobic conditions. Anaerobic fermentation was performed by adding 1% galacto-oligosaccharide (GOS), 1% MOS, and 2% MOS to the basal medium. Data are expressed as the mean ± standard deviation, and different letters indicate significant differences at *p* < 0.05 between groups at the same fermentation time.

Figure 4 illustrates the changes in the SCFA content during in vitro fermentation. When GOS and MOS were added, the most prominent SCFAs were acetic acid and propionic acid. The production amounts of acetic acid and propionic acid, which are major components of SCFAs, revealed a similar tendency when 1% GOS and 1% MOS were added (Figure 4A,B). However, the addition of 2% MOS indicated a different pattern. When 1% MOS and 1% GOS were added, the total SCFA content was maximum at 24 h, which then gradually decreased (Figure 4E). In contrast, the addition of 2% MOS gradually increased the total SCFA content as the fermentation time increased (Figure 4E). These results suggest that the fermentation time plays a crucial role in the treatment involving the addition of 2% MOS compared to those involving the addition of 1% MOS and GOS.

### 2.5. Species Richness and Diversity Following MOS and GOS Fermentations Using Fecal Microorganisms

Among the fecal microorganisms used for in vitro fermentation, the main microorganisms belonged to the phylum Firmicutes, followed by Actinobacteria, Proteobacteria, and Bacteroidetes (Figure S2). Microorganisms corresponded to the genus *Megamonas*, *Bifidobacterium*, and *Escherichia*. Unlike the MOS group, the GOS group revealed a high ratio in the *Lactobacillus* and *Megasphaera* genera.

**Figure 5:**
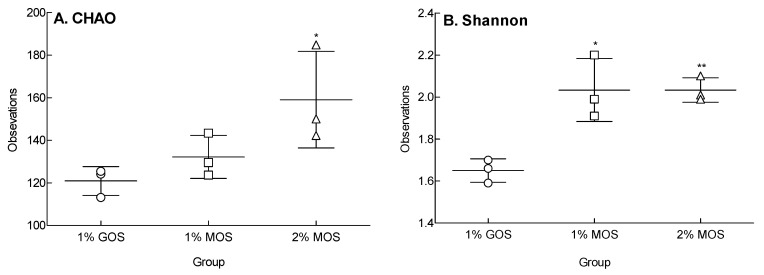
Alpha diversity of the three groups, 1% GOS, 1% MOS, and 2% MOS. The species richness (**A**) and species diversity (**B**) were evaluated by CHAO and Shannon indexes. The 1% GOS: Group fermented with fecal microbes by adding 1% galacto-oligosaccharide (GOS) to the basal medium; 1% MOS: Group fermented with fecal microbes by adding 1% malto-oligosaccharide (MOS) to the basal medium; 2% MOS: Group fermented with fecal microbes by adding 2% MOS to the basal medium. Data are expressed as the mean ± standard deviation, and different symbols indicate significant differences at * *p* < 0.05 and ** *p* < 0.01 vs. the GOS group.

Species richness and diversity were analyzed using the 16S rDNA sequences of the microorganisms (Figure 5). The operational taxonomic unit (OTU) values of each group were analyzed based on the similarity between the reads. There was a difference between the MOS and GOS groups. In particular, the 2% MOS group showed a significant increase in species richness (Figure 5A: *p* < 0.05) and diversity (Figure 5B: *p* < 0.01) compared to the group with the GOS group. The 1% MOS group did not present a significant difference in species richness compared to the GOS group, but revealed a significant difference in species diversity. Unlike GOS, MOS appeared to be involved in species richness, as well as diversity during fermentation.

### 2.6. Changes in Microbiota Following MOS and GOS Fermentation by Using Fecal Microorganisms

**Figure 6:**
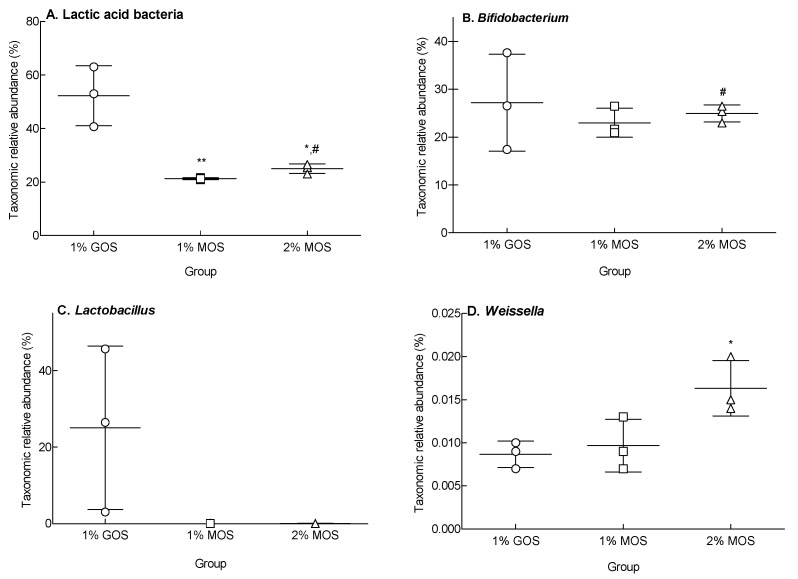
Relative abundance of lactic acid bacteria (**A**), *Bifidobacterium* (**B**), *Lactobacillus* (**C**), and *Weissella* (**D**) at the genus level. The 1% GOS: Group fermented with fecal microbes by adding 1% galacto-oligosaccharide (GOS) to the basal medium; 1% MOS: Group fermented with fecal microbes by adding 1% malto-oligosaccharide (MOS) to the basal medium; 2% MOS: Group fermented with fecal microbes by adding 2% MOS to the basal medium. Data are expressed as the mean ± standard deviation, and different symbols indicate significant differences at * *p* < 0.05 and ** *p* < 0.01 vs. the GOS group and ^#^ *p* < 0.05 vs. the MOS-1 group.

To evaluate the prebiotic activity of MOS, pyrosequencing was used to identify the diversity of bacterial populations after in vitro anaerobic fermentation (Figure 6). The relative abundance of total lactic acid bacteria (LAB), beneficial for the intestine, was significantly higher following GOS fermentation than MOS fermentation (1% MOS: *p* < 0.01, 2% MOS: *p* < 0.05). However, no significant difference was observed in the relative abundance of *Bifidobacterium* and *Lactobacillus*, which account for a larger proportion of total LAB, between GOS and MOS fermentation. The relative abundance of LAB in the 2% MOS group revealed a significant difference from that of the 1% MOS group (*p* < 0.05).

**Figure 7:**
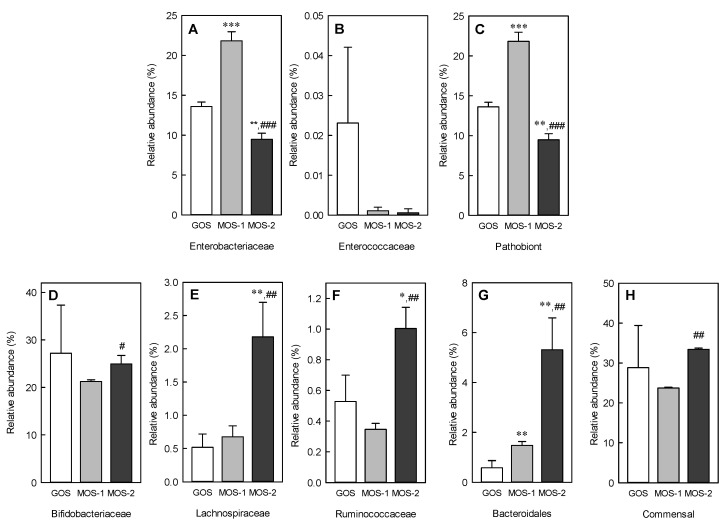
Relative abundance of *Enterobacteriaceae* (**A**), *Enterococcaceae* (**B**), total pathobiont strains (**C**), *Bifidobacteriaceae* (**D**), *Lachnospiraceae* (**E**), *Ruminococcaceae* (**F**), *Bacteroidales* (**G**), and total commensal strains (**H**) in the intestine at order and family levels. The total pathobiont strains are the sum of *Enterobacteriaceae* and *Enterococcaceae*, and the total commensal strains are the sum of *Bifidobacteriaceae, Lachnospiraceae, Ruminococcaceae*, and *Bacteroidales*. The 1% GOS: Group fermented with fecal microbes by adding 1% galacto-oligosaccharide (GOS) to the basal medium; 1% MOS: Group fermented with fecal microbes by adding 1% malto-oligosaccharide (MOS) to the basal medium; 2% MOS: Group fermented with fecal microbes by adding 2% MOS to the basal medium. Data are expressed as the mean ± standard deviation, and different symbols indicate significant differences at * *p* < 0.05, ** *p* < 0.01 and *** *p* < 0.001 vs. the GOS group and ^#^ *p* < 0.05, ^##^ *p* < 0.01, and ^###^ *p* < 0.001 vs. the MOS-1 group.

Additionally, the relative abundance of order-level Bacteroidales, known as commensal microbiota in the intestine, increased as the concentration of MOS increased (Figure 7). In the relative abundance of Bacteroidales, a significant difference was observed between the 2% and 1% MOS groups (Figure 7A,C: *p* < 0.01). The relative abundance of families Lachnospiraceae and Ruminococcaeceae was significantly increased in the 2% MOS group compared to GOS (Figure 7E: *p* < 0.01, Figure 7F: *p* < 0.05) and 1% MOS group (Figure 7E,F: *p* < 0.01). No significant difference was observed in the relative abundance of the commensal strains, including Bifidobacteriaceae, an important beneficial bacteria family present in the intestine, between GOS and MOS groups (Figure 7H). However, the 2% MOS group revealed a significant difference in the commensal strains compared to the 1% MOS group (Figure 7H: *p* < 0.01). Based on these results, the 2% addition of MOS appears effective in improving the intestinal microbiota and it can help maintain the health of the host owing to its prebiotic activity.

## 3. Discussion

Prebiotics such as FOS and GOS are known to selectively stimulate the growth and activity of healthy bacteria, including Bifidobacteria and lactic acid bacteria. The dietary fiber content of MOS prepared using only amylase was about 48%, of which it was reported to be 99% water-soluble [10]. To evaluate the prebiotic activity of MOS produced by α-amylase, the proliferation of *Bifidobacterium* as well as digestibility and fermentation characteristics of fecal microorganisms were evaluated via in vitro tests.

MOS is an oligosaccharide produced by glycosyltransferase and glycosylhydrolase, and it comprises 2–10 α-d-glucopyranosyl units linked only by α-1,4 glycosidic bonds. However, MOS produced by thermophilic α-amylase comprises oligomers up to DP5 (Table 1). MOS produced by the α-amylase treatment presents alpha-1-6 glucosyl linkage, unlike the MOS treated with pullulanase. α-Amylase produces limit α-dextrins, short linear oligosaccharides, and glucose [18]. The α-amylase reaction leaves a certain amount of limit dextrin in the product [6]. Recently, it has been reported that indigestible dextrin, α-cyclodextrin, and dextran increase intestinal SCFA production [19]. According to our previous studies, approximately 48% of dietary fiber in MOS prepared using amylase only was 99% water-soluble and can be used as prebiotics [10].

In order to evaluate the prebiotic activity of MOS produced only by amylase, it is necessary to evaluate the proliferative activity of MOS against Bifidobacteria, which is a beneficial bacteria in the intestine, and the digestibility of MOS against digestive enzymes. MOS significantly increased the proliferation of *B. breve* compared to the known prebiotics FOS and GOS, and similar results were obtained with FOS in the proliferation of *B. longum* (Figure 1). *Bifidobacterium* has been reported to improve constipation and enhance immunity by inhibiting the growth of pathogenic microorganisms that produce harmful substances in the intestine [20]. MOS significantly increased the proliferation of *B. breve* compared to the known prebiotics FOS and GOS, and similar results were obtained with FOS in the proliferation of *B. longum* (Figure 1). As MOS is produced by the amylase treatment, it is resistant to hydrolysis by salivary α-amylase and is converted to DP1–DP3 by hydrolysis of DP5 and DP4 saccharose only by intestinal glucoamylase. In the mouth, long-chain carbohydrates are partially digested by the enzymatic action of salivary α-amylase, the main enzyme responsible for hydrolysis. Structures resistant to salivary amylase commonly include α-1,6 linkages or glucosyl residues around α-1,6 linkages [21].

To evaluate the effect of MOS, which showed the proliferative activity of *Bifidobacterium*, on the improvement of the intestinal microbiota, in vitro fermentation was performed by the mixed fecal microorganisms derived from humans. In Figure 4, 1% MOS added to the basal medium increased the production of SCFA by the fecal microorganisms compared to 1% GOS and 2% MOS. According to Sako et al. [22], the amount and type of SCFAs produced in the intestine may vary depending on the substrate, as well as the composition of the intestinal microbiota. Moreover, the utilization rate of oligosaccharides may depend on the degree of polymerization, degree of glycoside binding and branching, synergy between bacteria during fermentation, relationship between bacteria and fermentation products, characteristics of fermentation, and the ability to decompose sugars [23]. Acetate with the highest SCFA content, produced by intestinal microbes using these oligosaccharides, is important for suppressing pathogenic bacteria in the intestine by lowering the pH [24].

The population of commensal bacteria during fermentations largely depends on indigestible carbohydrates that are not used by the host into SCFAs in the intestine [25]. Commensal bacteria also enhance immunity by suppressing pathogenic bacteria in the intestine. *Enterobacteriaceae*, *Fusobacteriaceae*, and *Enterococcaceae*, belong to *Proteobacteria*, and are known to induce inflammation. In contrast, strains corresponding to commensal, *Bacteroidales*, *Lachnospiraceae*, *Ruminococcaceae*, and *Bifidobacteriaceae* inhibit the proliferation of pathogenic bacteria [26]. No significant difference was observed in the intestinal resident bacteria between the GOS and MOS groups. However, the relative abundance of pathogenic bacteria was significantly lower in the 2% MOS group than in the GOS group (Figure 7). Furthermore, the addition of 2% MOS, similar to GOS, could selectively enhance the proliferation of commensal bacteria, which is believed to inhibit the growth of pathogenic bacteria in the intestine.

MOS, which is involved in the proliferation of commensal bacteria, exhibited the increase of species diversity and richness during in vitro fermentation, indicating the intestinal utilization of MOS (Figure 5). An increase in the relative abundance of *Bifidobacterium* represents intestinal health, thus revealing the beneficial effects of this species on colon cancer, colon regularity, and acute diarrhea [27,28]. As illustrated in Figure 6, GOS contributed to the proliferation of *Bifidobacterium* and *Lactobacillus* as a typical prebiotic and significantly increased the relative abundance of total LAB compared to the MOS groups (*p* < 0.05). In contrast, the addition of 2% MOS selectively contributed to the proliferation of *Bifidobacterium* compared to that of 1% MOS, thereby adding to the total LAB proliferation. While the intake of prebiotics was associated with increased microbial diversity [29], the diversity of intestinal microflora was reduced by *Clostridium* sp. infection, Crohn’s disease, and obesity [29,30]. When mice were orally administered with high purity GOS, the intestinal microbial diversity and species richness were significantly increased [31].

In conclusion, MOS increased the species diversity and richness of intestinal microbiota during in vitro fermentation using human fecal microbiota. In addition, MOS has shown potential as a prebiotic with increased SCFA production and enhanced growth of beneficial bacteria during fermentation. Due to differences in intestinal microbiota between different populations, individual fecal cultures of populations will differ from mixed cultures in terms of changes in intestinal strains. It is necessary to evaluate the prebiotic potential of MOS in vivo with experimental animals in order to precisely assess its effect on the intestinal microbiota. Therefore, we intend to measure the effect of MOS in enhancing the intestinal microbial growth through further animal experiments.

## 4. Materials and Methods

### 4.1. MOS Production

Batch reactions were carried out by incubating α-amylase with a 40% rice powder suspension in a 100-L incubator shaker at 150 rpm. Rice powder (20 kg) was suspended in water (50 L) and α-amylase was added in an amount equivalent to 2% of the substrate. For liquefaction and saccharification, MTAA (Shandong Longda Bio-Products Co., Ltd., Yishui, China) and Fungamyl 800L (Novozymes, Bagsvaerd, Denmark) were used, respectively, whereas HTAA (Shandong Longda Bio-Products Co., Ltd.) was used as an enzyme that could react without distinguishing between liquefaction and saccharification The enzyme reaction proceeded for approximately 24 h at 90 °C and was terminated by a heat treatment at 100 °C for 15 min. MOS was filtered and treated with activated carbon for decolorization. The MOS syrup was concentrated under reduced pressure to 75° brix at 60 °C. The MOS production was carried out in three batches, and MOS production data based on the differences in enzymes and treatment processes were expressed using the results of each experiment.

### 4.2. Evaluation of MOS Availability by Bifidobacterium sp.

In order to measure the sugar availability by *Bifidobacterium* sp., 1% GOS, FOS, or MOS were added to the modified peptone yeast extract fructose (PYF) medium without a carbon source. *B. bifidum* KCTC 3357, *B. longum* SJ 32, and *B. breve* ATCC 15,700 were inoculated in the sterilized medium and incubated in an anaerobic condition at 37 °C, and the samples were collected at intervals of 12 h. The absorbance of the collected samples was measured at 600 nm to evaluate the degree of bacterial growth.

### 4.3. In Vitro Digestion of MOS

The in vitro digestion of MOS was examined using oral, gastric, and pancreatic enzymes. To measure the digestibility in the mouth, 600 mg of MOS was dissolved in a buffer solution (KCl 15.1 mmol/L, KH_2_PO_4_ 3.7 mmol/L, NaHCO_3_ 13.6 mmol/L, MgCl_2_(H_2_O)_6_ 0.15 mmol/L, (NH_4_)_2_CO_3_ 0.06 mmol/L, CaCl_2_(H_2_O)_2_ 1.5 mmol/L, pH 7.0), and then 120 μL of salivary amylase (1500 U/mL, human saliva type IX-A) was added. After 30 min, the enzyme was inactivated by a heat treatment at 95 °C for 10 min. After adjusting the pH to 2.0 with HCl (0.2 mol/L), pepsin from the porcine gastric mucosa (15.750 units EC 3.4.23.1) was added and gastric digestion was performed at 37 °C for 2 h. Thereafter, gastric digestion was terminated by adjusting the pH to 7.0 with NaHCO_3_. For pancreatic digestion, pancreatic α-amylase and glucoamylase (pancreatin from porcine pancreas, Sigma-Aldrich, St. Louis, MO, USA) were added to MOS dissolved in the intestinal buffer (KCl 6.8 mmol/L, KH_2_PO_4_ 0.8 mmol/L, NaHCO_3_ 85 mmol/L, NaCl 38.4 mmol/L, MgCl_2_(H_2_O)_6_ 0.33 mmol/L, CaCl_2_(H_2_O)_2_ 0.6 mmol/L, pH 7.0) and reacted at 37 °C for 2 h. The pancreatic digestion was inhibited by heating at 100 °C for 10 min.

### 4.4. In Vitro Fecal Fermentation

In vitro fecal fermentation was performed in three replicates under anaerobic conditions using a 100-mL serum bottle [26]. The basal medium components used were as follows: 2 g/L peptone water, 2 g/L yeast extract, 0.1 g/L NaCl, 0.04 g/L K_2_HPO_4_, 0.04 g/L KH_2_PO_4_, 0.01 g/L MgSO_4_(H_2_O)_7_, 0.01 g/L CaCl_2_(H_2_O)_6_, 2 g/L NaHCO_3_, 2 mL Tween 80, 0.02 g/L hemin, 10 mL vitamin K1, 0.5 g/L cysteine HCl, and 0.5 g/L bile salts. MOS was added to the basal medium at final concentrations of 1% and 2%. The medium was adjusted to pH 6.8, and after adding resazurin (1 mg/L), it was replaced with oxygen-free nitrogen gas to maintain anaerobic conditions. Fresh fecal samples were stored in sterile fecal containers with gas park generators (Becton Dickinson, Sparks, MD, USA) within 10 min after collection from three healthy adult donors (one per donor, at least 0.5 g) to preserve the viability of anaerobic bacteria. Feces were homogenized for 5 min by BOSCH Ultracompact in PBS (100 mM, pH 7.0) and were inoculated with an amount equivalent to 2% of the medium volume. Since a large difference was observed in the fecal colonies between individuals, three repeated experiments were conducted by mixing the feces of three individuals to minimize the difference.

After incubating at 37 °C, the sample was collected at intervals of 12 h to measure changes in the components during fermentation. Moreover, as a positive control, 1% GOS (Neo Crema Co., Ltd., Seoul, Korea) was added to the basal medium to proceed with fermentation. All the medium components and chemicals were purchased from Sigma-Aldrich.

The experimental protocol was approved by the Jeonju University Institutional Review Board (jjIRB-201013-BR-2020-1036) and was conducted in accordance with the ethical standards of 1964 Declaration of Helsinki. Fresh stool samples were obtained with written consent from healthy men in their 20s and 30s who did not receive antibiotics or pre/probiotics from 3 months prior to the experiment and did not have a history of smoking and gastrointestinal disorders. Experiments were commenced within 2 h of stool collection.

### 4.5. Assay of MOS

A Waters HPLC system comprising an ELS detector (Waters, Wexford, Ireland) was used to detect MOS. MOS was separated on an NH_2_P-50 4E amine column (250 × 4.6 mm ID) using 64% acetonitrile as the mobile phase at a flow rate of 1.0 mL/min [32]. DP1 (glucose) and DP2 (maltose), which were used as standards, were purchased from Sigma-Aldrich and DP3 (maltotriose), DP4 (maltotetraose), and DP5 (maltopentaose) were purchased from Elicityl-oligotech (Crolles, France).

### 4.6. Assay of SCFAs

The SCFA analysis was conducted according to a previous study report [10]. Samples (2 mL) were collected from the culture medium using a sterile syringe at 12 h intervals and stored at −80 °C until the SCFA analysis. Briefly, the samples were centrifuged (5000× *g*) at room temperature for 15 min, and the supernatant was used for analysis. Gas chromatography (Agilent Technologies, Santa Clara, CA, USA) was equipped with a GC column (DBFFAP 123-3253, 50 m × 0.32 mm × 0.50 μM), flame ionization detector, and an autosampler. The injector and detector port temperatures were 200 and 240 °C, respectively. The carrier gas was N_2_ at a flow rate of 1.4 mL/min. The SCFA concentration was expressed in mM.

### 4.7. Microbial Analysis of MOS Fermentation Broth by Fecal Microorganisms

In order to investigate the effect of MOS on changes in the intestinal microorganisms, fresh fecal samples were inoculated with MOS and glucose in anaerobic conditions for 36 h and then stored at −80 °C to isolate genomic DNA (gDNA). Intestinal microbial gDNA was extracted using a DNA MiniPrep kit (Zymo Research, CA, USA) and stored at −20 °C until further use. Microorganisms in the culture medium were analyzed for 16S rDNA gene sequences using the pyrosequencing method [33]. The base sequences obtained through pyrosequencing were analyzed using the CLcommunityTM software (Chunlab Inc., Seoul, Korea), and the species were defined by a 97% sequence similarity using the CD-HIT program and the OTU values [34]. Species richness was expressed by alpha-diversity, an indicator of biological species diversity, and species abundance was indicated by analyzing CHAO 1 richness [35] and the Shannon diversity index [36].

### 4.8. Statistical Analysis

All measured values were expressed as means and standard deviations. The statistical analysis between each experimental group was performed by a one-way analysis of variance (ANOVA) with Tukey’s multiple comparison tests using SPSS (SPSS; ver. 12.0, SPSS Inc., Chicago, IL, USA). A *p*-value of less than 0.05 was considered statistically significant between groups.

## Figures and Tables

**Table 1:** Malto-oligosaccharide (MOS) production by different enzyme and treatment processes.

Process	Time (h)	Maltotriose (%) (DP3)	Maltotetraose (%) (DP4)	Maltopentaose (%) (DP5)	Total MOS (%)
Liquefaction by MTAA	4	3.32 ± 0.45	2.43 ± 0.32	2.16 ± 0.26	7.91 ± 1.03
Saccharification by Fungamyl 800L	8	5.23 ± 0.32	4.64 ± 0.64	3.83 ± 0.35	13.70 ± 1.31
	13	8.27 ± 0.47	7.36 ± 0.57	6.99 ± 0.75	22.62 ± 1.79
	20	14.21 ± 1.24	12.11 ± 1.04	9.24 ± 1.01	35.56 ± 3.29
	24	14.50 ± 0.45	12.58 ± 0.85	9.63 ± 0.84	36.71 ± 2.14
Simultaneous liquefaction and saccharification by HTAA	4	5.83 ± 0.33	5.41 ± 0.38	3.53 ± 0.33	14.77 ± 1.04
	8	9.20 ± 0.45	7.95 ± 0.59	10.11 ± 0.54	27.26 ± 1.58
	13	14.77 ± 0.75	13.23 ± 0.65	10.93 ± 0.85	38.93 ± 2.25
	20	21.74 ± 1.12	18.84 ± 0.83	11.76 ± 0.93	52.34 ± 2.88
	24	21.54 ± 1.01	17.32 ± 0.73	12.83 ± 0.56	51.69 ± 2.30

Data are expressed as the mean ± standard deviation (SD) from triplicate experiments.

## Supplementary Materials

The following are available online. Figure S1: Changes in the pH and reducing sugar content of malto-oligosaccharide (MOS) by in vitro fermentation; Figure S2: Relative abundance of microbial communities at the level of phylum and genus in three groups, GOS, MOS-1, and MOS-2.
