# Supplementary material for: In Vitro Prebiotic Effects of Malto-Oligosaccharides Containing Water-Soluble Dietary Fiber
Source: Molecules. 2020 Nov 9;25(21):5201. doi: 10.3390/molecules25215201 (PMC7664926; doi:10.3390/molecules25215201)
Supplement: Supplementary file 1 [file molecules-25-05201-s001.pdf]

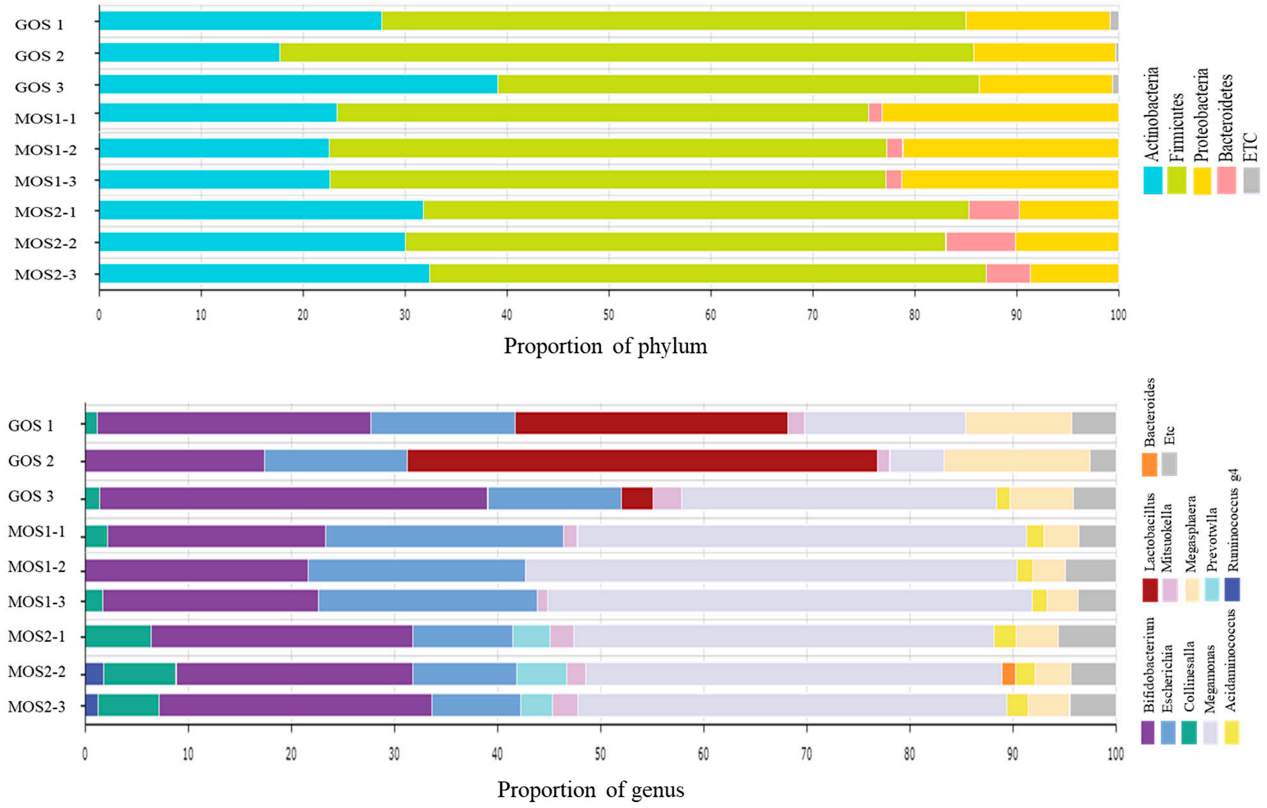

Supplementary 1. Relative abundance of microbial communities at the level of phylum and genus in three groups, GOS, MOS-1, and MOS-2. GOS: Group fermented with fecal microbes by adding 1% galacto-oligosaccharide (GOS) to basal medium; MOS-1: Group fermented with fecal microbes by adding 1% malto-oligosaccharide (MOS) to basal medium; MOS-2: Group fermented with fecal microbes by adding 2% MOS to basal medium.
